# High-density SNP linkage map construction and QTL mapping for flavonoid-related traits in a tea plant (*Camellia sinensis*) using 2b-RAD sequencing

**DOI:** 10.1186/s12864-018-5291-8

**Published:** 2018-12-22

**Authors:** Li-Yi Xu, Li-Yuan Wang, Kang Wei, Li-Qiang Tan, Jing-Jing Su, Hao Cheng

**Affiliations:** 1 National Centre for Tea Improvement, Tea Research Institute, Chinese Academy of Agricultural Sciences, Hangzhou, 310008 China; 2 College of Horticulture and Forestry Science, Huazhong Agricultural University, Wuhan, 430070 China; 3 College of Horticulture, Sichuan Agricultural University, Chengdu, 611130 China

**Keywords:** Genetic map, SNP, QTL mapping, 2b-RAD sequencing

## Abstract

**Background:**

Flavonoids are important components that confer upon tea plants a unique flavour and health functions. However, the traditional breeding method for selecting a cultivar with a high or unique flavonoid content is time consuming and labour intensive. High-density genetic map construction associated with quantitative trait locus (QTL) mapping provides an effective way to facilitate trait improvement in plant breeding. In this study, an F1 population (LJ43×BHZ) was genotyped using 2b-restriction site-associated DNA (2b-RAD) sequencing to obtain massive single nucleotide polymorphism (SNP) markers to construct a high-density genetic map for a tea plant. Furthermore, QTLs related to flavonoids were identified using our new genetic map.

**Results:**

A total of 13,446 polymorphic SNP markers were developed using 2b-RAD sequencing, and 4,463 of these markers were available for constructing the genetic linkage map. A 1,678.52-cM high-density map at an average interval of 0.40 cM with 4,217 markers, including 427 frameset simple sequence repeats (SSRs) and 3,800 novel SNPs, mapped into 15 linkage groups was successfully constructed. After QTL analysis, a total of 27 QTLs related to flavonoids or caffeine content (CAF) were mapped to 8 different linkage groups, LG01, LG03, LG06, LG08, LG10, LG11, LG12, and LG13, with an LOD from 3.14 to 39.54, constituting 7.5% to 42.8% of the phenotypic variation.

**Conclusions:**

To our knowledge, the highest density genetic map ever reported was constructed since the largest mapping population of tea plants was adopted in present study. Moreover, novel QTLs related to flavonoids and CAF were identified based on the new high-density genetic map. In addition, two markers were located in candidate genes that may be involved in flavonoid metabolism. The present study provides valuable information for gene discovery, marker-assisted selection breeding and map-based cloning for functional genes that are related to flavonoid content in tea plants.

**Electronic supplementary material:**

The online version of this article (10.1186/s12864-018-5291-8) contains supplementary material, which is available to authorized users.

## Background

Tea plants [*Camellia sinensis* (L.) Kuntze] are the raw materials for one of the three most popular non-alcoholic drinks in the world [1, 2]. Because of its high economic value, it has been extensively planted in China, India, Sri Lanka and so on [3, 4]. Therefore, selecting new tea cultivars with improved production and quality characteristics has received much attention in these countries. However, compared with other crops, tea plants have a high self-incompatibility and require more than three years to grow and develop, which makes it difficult to carry out genetic improvement [5], while traditional breeding methods are still commonly used. A genetic linkage map, particularly a high-density map, plays a major role in plant breeding as a fundamental tool for genetic analysis and further functional gene localization. Such maps are based on calculating the recombination rates between many molecular markers [6–8]. Thus, constructing genetic maps and further quantitative trait locus (QTL) analysis should be effective methods to facilitate trait improvement in breeding tea cultivars.

With advances in DNA sequencing technology, molecular marker technologies, especially SNPs (single nucleotide polymorphisms), play a major role in breeding [9–11]. Traditionally, randomly amplified polymorphic DNA (RAPD), amplified fragment length polymorphism (AFLP), restriction fragment length polymorphism (RFLP) and simple sequence repeats (SSRs) have been used to construct genetic maps and identify QTLs in plants [12, 13]. However, they have a low density and uneven distribution throughout the genome, which restricts the accuracy of QTL analysis [14]. SNP markers based on high-throughput sequencing for whole or partial genomes have a high density and even distribution in genomes, and they have been rapidly developed as new molecular markers [15]. 2b-Restriction site-associated DNA (2b-RAD) sequencing is a high-throughput sequencing method that uses type IIB restriction endonucleases to produce uniform fragments for sequencing. Benefiting from the character of type IIB restriction endonucleases, these fragments are of the same length and have high coverage in chromosomes, which make them particularly suitable for high-throughput genotyping to construct genetic linkage maps in natural populations [16]. Therefore, in this study, 2b-RAD sequencing was chosen to develop SNP markers to construct a high-density genetic map for a tea plant.

Based on genotyping for molecular markers, some genetic maps have recently been constructed by using various types of markers in tea plants. For example, Chang et al. [17] constructed a genetic map using 678 markers including 143 RAPDs, 11 public SSRs, 495 AFLPs, and 29 newly mined SSRs, and the map covered 1441.6 cM with an average distance of 4.7 cM between adjacent markers. Ma et al. [18] developed an SSR genetic map containing 406 SSRs spanning 1143.5 cM with an average distance of 2.9 cM between markers. Then, they re-constructed a SNP/SSR genetic map with 6,042 SNPs and 406 SSRs distributed over 3,965 cM with an average distance of 1.0 cM between markers using specific length amplified fragment (SLAF)-seq [19]. However, to our knowledge, the average distance between markers is still more than 1.0 cM in all published genetic maps for tea plants. Thus, we attempted to construct a denser genetic map by using large SNP markers based on 2b-RAD sequencing to improve the precision of QTL analysis and reduce the difficulty of fine QTL mapping.

In general, major secondary metabolites containing flavonoids, caffeine and amino acids are important components of tea extraction that provide the rich flavours, fresh taste and nutrient content of tea products [20]. Among them, flavonoids are the most abundant component, are driven by phenylalanine and include flavones, flavonols, isoflavones, flavanones, flavanols, and anthocyanidins [21]. In flavonoids, catechin components are the most prevalent, and their ability to effectively react with oxygen has proven them to be scavengers [22]. The variation in catechins in young leaves is responsible for the various quality and flavour characteristics during tea production. The lower levels of catechins can make green tea to taste less astringent and more umami [23, 24]. In addition, flavonoids with a wide range of functions, for example, antioxidant activity, ultraviolet light protection, and defence against phytopathogens, were also found [25]. However, only one QTL region related to the catechin content, timing of spring bud flush, young shoot colour (YSC) and mature leaf size has been identified in the SSR genetic map [18, 26]. Due to a small mapping population or few markers in the genetic map, some maps may be unsuitable for QTL mapping. Thus, it is necessary to construct a high-density SNP genetic linkage map and identify the QTLs with flavonoid-related traits for further functional gene discovery and molecular marker-assisted breeding of tea plants.

In this study, the aims were 1) to construct a high-density genetic map using 2b-RAD sequencing, 2) to map QTLs for flavonoid-related traits in the F1 group, and 3) to select candidate genes that may affect the target trait via sequence comparison and functional annotation.

## Methods

### Plant materials and DNA extraction

The F1 segregating population consisted of 327 individuals was derived from an inter-varietal cross between two clonal tea cultivars, *C. sinensis* cv. *Longjing 43* (‘LJ43’) and *C. sinensis* cv. *Baihaozao* (‘BHZ’). ‘LJ43’ was used as the female parent. All individuals were planted at the germplasm resources of the Tea Research Institute in the Chinese Academy of Agricultural Sciences in Hangzhou, Zhejiang, China. Detailed information on the F1 population was published in Tan *et al*. [8]. Fresh and tender leaves from each of the 327 progenies and their parents were collected to extract genomic DNA using a Tiangen Plant DNA Kit DP305 (Beijing, China). The quality and concentration of the extract products were detected using a Nanodrop ND-2000 (Thermo Scientific, USA), and they were preserved at −20°C.

### Library preparation, sequencing and de novo genotyping

The genomic DNA of the 327 progenies and two parents were used to construct 2b-Rad libraries using type IIB restriction enzymes, following the protocol developed by Wang *et al*. [16]. First, 100-200 ng of genomic DNA was digested at 37°C for 3 h in a 15-μl reaction that contained 4 U of BsaXI. Then, the digestion product was incubated at 4°C for 16 h in a 12-μl ligation master mix consisting of 0.2 μM library-specific adaptors, 1 mM ATP and 800 U of T4 DNA ligase. In detail, after type IIB restriction enzyme (BsaXI) digesting, 33bp tags (5’-NNNNNNNNNACNNNNNCTCCNNNNNNNNNn-3’) were obtained. Among the tag, ‘NNn’ of 3’ sticky end could be paired with special adaptors consisted of 6 bp recognition base and 3 bp overhang (Additional file 1: Table S1). With changing of base, various overhang-types were formed, for instance, the 5’-NNN-3’ overhang containing a total of 64 overhang-types while only 16 overhang-types in 5’-NNA-3’ or 5’-NNT-3’ overhang-type. We could change the last base of overhang to reduce the density of tags to save cost. In our study, adaptors with 5’-NNN-3’ overhang were used to construct the standard BsaXI libraries of two parents, whereas adaptors with 5’-NNA-3’ and 5’-NNT-3’ overhangs were constructed for each progeny to reduce the representation library. In the second PCR amplification, a specific adapter barcode was incorporated into each library; then, all libraries were pooled for single-end sequencing (1 × 50 bp) using an Illumina HiSeq 2000 sequencer.

De novo genotyping followed the analytical approach described by Jiao *et al*. [27]. Briefly, reads with the following characteristics were removed: no restriction sites, long homopolymers (more than 10 bp) and low quality (more than 10 bases with a quality fraction of < 20). RADtyping program v. 1.3, which is an integrated package for accurate de novo codominant and dominant RAD genotyping in mapping populations, was used for the de novo 2b-RAD genotyping using the default parameters [28].

### 2b-RAD data analysis

The markers were genotyped according to the RAD-typing approach for the non-reference genomes [29]. In general, the markers of the two parents were used to construct a reference sequence. Then, the high-quality reads of the progenies were mapped to a high-quality reference sequence and clustered to obtain unique tags for genotyping by SOAP software (http://soap.genomics.org.cn) [29]. Unique markers that had a sequencing depth of less than 3 were removed.

### Genetic linkage mapping

Markers that could be genotyped in at least 80% of the progenies were chosen for further analysis. The genetic maps were constructed by high-quality SNP markers that conformed to the Mendelian separation ratio (p ≥ 0.05) via the χ2 test and a set of 483 SSR markers previously mapped in this population [30].

First, sex-specific maps were constructed for each parent using the two-way pseudo-testcross strategy [31]. Female and male datasets were created using the command ‘create maternal and paternal population nodes’ in the JoinMap 4.0 program [32]. The heterozygous markers in either the female or the male were tagged as ‘f’ of ‘m’, and bi-markers were tagged as ‘h’, whereas the dominant markers were tagged as ‘df’ or ‘dm’. Markers were grouped at a logarithm of odds (LOD) threshold value of 5.0. The regression mapping algorithm was used for map construction. Marker distances in cM were calculated using Kosambi’s mapping function. Linkage groups were drawn visually using the MapChart software [33]. A combined map was estimated by integrating the female and male maps using the shared markers in MergeMap software [34]. Two total genetic map lengths were estimated based on the combined map, Ge1 [35] and Ge2 [36], and their average was used as the predicted total genetic map length (Ge). The genome coverage (Cof) was calculated by Gof/Ge, where Gof was the observed genetic map length (the sum of the map lengths of all linkage groups).

### Phenotype data collection

Data for ten traits were collected for the QTL analysis, which contained the anthocyanin content (OPC), YSC, caffeine content (CAF) and seven components of catechins (GC, EGC, C, EC, EGCG, GCG and ECG). The YSC data came from our previous studies [30]. In this study, the CAF and the seven components of catechins in each mapping individual were measured using HPLC in 2014 and 2015, respectively. This spectrophotometric method was used to measure the OPC, which included the following steps: first, young shoots of each mapping individual were picked and freeze dried. Then, the OPC was extracted from the tea sample via incubation in a 0.1 mol/L hydrochloric acid ethanol solution at 60 °C for 1 h. Finally, optical density values at 530 nm, 620 nm and 650 nm were measured to calculate the OPC. Repeat the process for each test and average the results.

### QTL mapping analysis

QTL mapping analysis was performed for ten traits based on the combined linkage map in MapQTL 5 software [37]. In general, the LOD scores were analysed using the interval mapping method; then, the genome-wide and chromosome-wide LOD significance thresholds at the 95% level were determined via a 1,000-permutation test for each trait. QTLs with LOD scores greater than the chromosome threshold at 95% were declared significant. Finally, all QTLs were named according to Cui et al. [38].

### SNP annotation

The mapped markers were aligned against the reference genome [39] using SOAP software. The parameters were set to (-r 0, -M 0), ‘-r 0’ meaning no repeats and ‘-M 0’ meaning no mismatch. After removing the unaligned marker, the markers that were mapped in the QTL regions were annotated to ensure the position and function in the reference genome using SnpEff 4.1 software.

## Results

### 2b-RAD library

The 2b-RAD library of the 327 individuals and two parents generated a total of 1,002,035,680 reads (Table 1). The average number of reads of the standard library consisting of two parents was 40,833,712, whereas that of each progeny was 2,814,582, which used selective overhangs (5’-NNA-3’ and 5’-NNT-3’). After sequence filtering and removing the low-quality reads, more than 85% of the 317 progeny libraries contained high-quality reads with a restriction site (Additional file 1: Table S1). After mapping the high-quality reads of the progenies into reference sequences, each progeny obtained 71,852 unique markers, and the sequencing depth reached an average of 20x, whereas there were 190,468 unique markers for each parent in which the sequencing depth reached an average of 123x (Additional file 2: Table S2).

**Table 1:** The summaries of high-throughput sequencing

Sample	Original reads	High-quality reads	Ratio (%)
LJ43	44,091,387	40,487,516	91.8
BHZ	37,576,037	33,514,594	89.2
327 Progenies	920,368,256	797,734,136	86.7
Total	1,002,035,680	871,736,246	87.0

### SNP markers

**Fig. 1:**
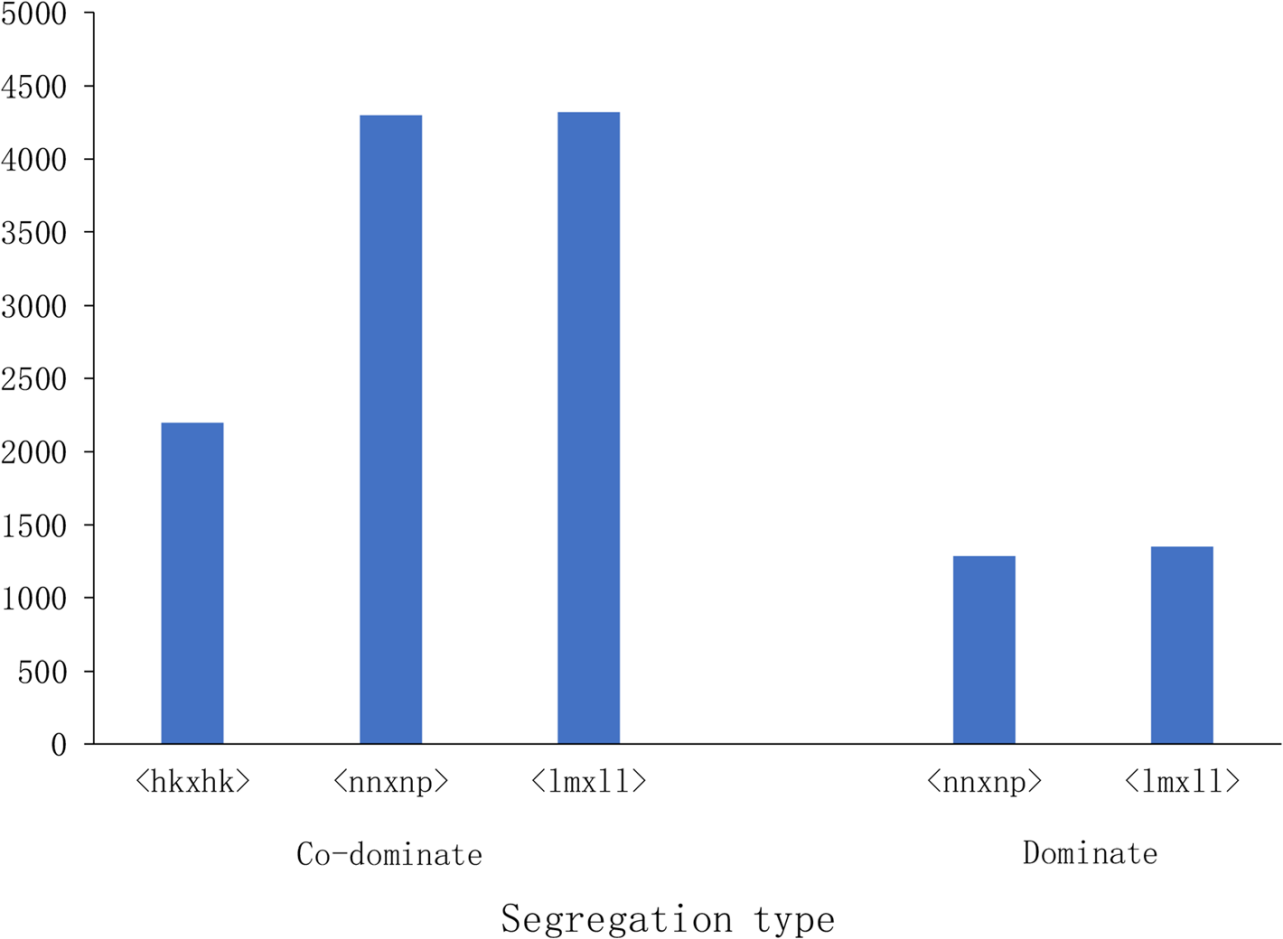
Number of SNPs for segregation type (Co-dominate markers consists of three segregation types: hk×hk, lm×ll, nn×np; dominate markers consists of two segregation types: lm×ll, nn×np)

A total of 46,932 2b-RAD loci with different genotypes in the two parents were detected (Additional file 3: Table S3). After filtering, both had low- or high-coverage locus clusters due to sequencing errors or repeat sequences, respectively, and SNP genotyping was performed. A total of 10,816 co-dominant and 2,630 dominant 2b-RAD markers were detected (Additional file 4: Table S4). Among these markers, three segregation types were classified, including hk×hk, lm×ll and nn×np (Fig. 1). Moreover, 2b-RAD markers with genotyping information in more than 80% of F1 individuals were selected. After the χ2 test, a total of 3,026 co-dominant and 1,437 dominant 2b-RAD markers were available to construct the genetic linkage map. Among these markers, 77.7% were heterozygous in the female parent, whereas 74.0% were heterozygous in the male parent.

### Genetic linkage map

In this study, a total of 4,943 markers were used to construct the genetic linkage map, which consisted of 4,463 newly developed SNPs and 483 published SSRs. The SSR markers could be used to ensure the frame of the genetic linkage map. After genetic mapping using JoinMap 4.0, for both the female and male genetic maps, the markers were divided into fifteen linkage groups at a LOD threshold of 5.0. In the female genetic map, the linkage length ranged from 73.49 to 144.17 cM with 82 to 290 markers. In total, the female map included 2,470 markers spanning 1427.70 cM with an average interval of 0.58 cM. In the male genetic map, the linkage length ranged from 78.71 to 161.20 cM with 76 to 208 markers. In total, the male map included 2,217 markers spanning 1710.63 cM with an average interval of 0.78 cM (Additional file 5: Table S5).

Furthermore, by integrating the female and male maps using the shared markers, a high-density map with 4,217 markers mapped into 15 linkage groups, including 427 SSRs and 3,800 SNPs, was successfully constructed (Table 2, Additional file 6: Figure S1). The combined map spanned a total of 1,678.52 cM with an average marker distance of 0.40 cM and an average of 281 markers in each linkage group  (Additional file 7: Table S8). The genetic lengths of the 15 linkage groups ranged from 87.46 to 146.93 cM, with an average marker distance of 0.30 to 0.48 cM in the fifteen linkage groups. The longest and largest linkage group was LG03, with a genetic distance of 146.93 cM and 374 markers, whereas the shortest was LG15, which spanned 87.46 cM and contained 223 markers. The smallest group was LG12, with 206 markers. The integrity of the combined map with gaps of less than 2 cM between each adjacent marker ranged from 95.71% (LG04) to 98.92% (LG03), with an average of 98.19%. The integrity of the 14 linkage groups was greater than 98%, except for that of LG04, indicating the comparatively high coverage and integrity in these combined maps.

**Table 2:** Information on the genetic map

Linkage group	Markers no.			Distance ^a^ (cM)	Length (cM)	Gaps < 2 cM (%)	Max Gap (cM)
	SSR	SNP	Total				
LG01	40	331	371	0.40	146.61	97.83	5.10
LG02	33	248	281	0.44	123.96	98.21	5.40
LG03	38	336	374	0.39	146.93	98.92	3.79
LG04	8	226	234	0.48	111.71	95.71	5.75
LG05	16	325	341	0.30	103.51	98.24	6.11
LG06	31	276	307	0.40	122.31	98.04	6.03
LG07	29	232	261	0.40	103.45	98.08	8.70
LG08	42	265	307	0.41	125.03	98.04	4.57
LG09	34	310	344	0.37	126.89	98.83	3.42
LG10	27	242	269	0.37	99.58	98.51	2.58
LG11	30	225	255	0.39	98.46	98.03	5.33
LG12	23	183	206	0.45	92.57	98.54	6.99
LG13	20	201	221	0.44	96.74	98.64	8.89
LG14	27	196	223	0.42	93.31	98.65	4.87
LG15	17	206	223	0.39	87.46	98.65	2.96
Total	417	3800	4217	0.40	1678.52	98.19	8.89

^a^Indicates the average interval between adjacent markers in each LG

Moreover, to compare the map alignment and ensure the map orientation, the 427 shared SSR markers on our map were used to align the SSR-based genetic map constructed in our previous study [30]. Overall, we successfully aligned our genetic linkage map (Map1) with the SSR-based genetic map (Map2) at a one-to-one correspondence (Additional file 8: Figure S2). A relatively consistent order was observed for almost all linked markers between the two maps. However, only 3 markers changed over the long distance between linked markers in LG03, LG08 and LG10.

### Phenotypic statistical analysis of flavonoid-related traits

**Fig. 2:**
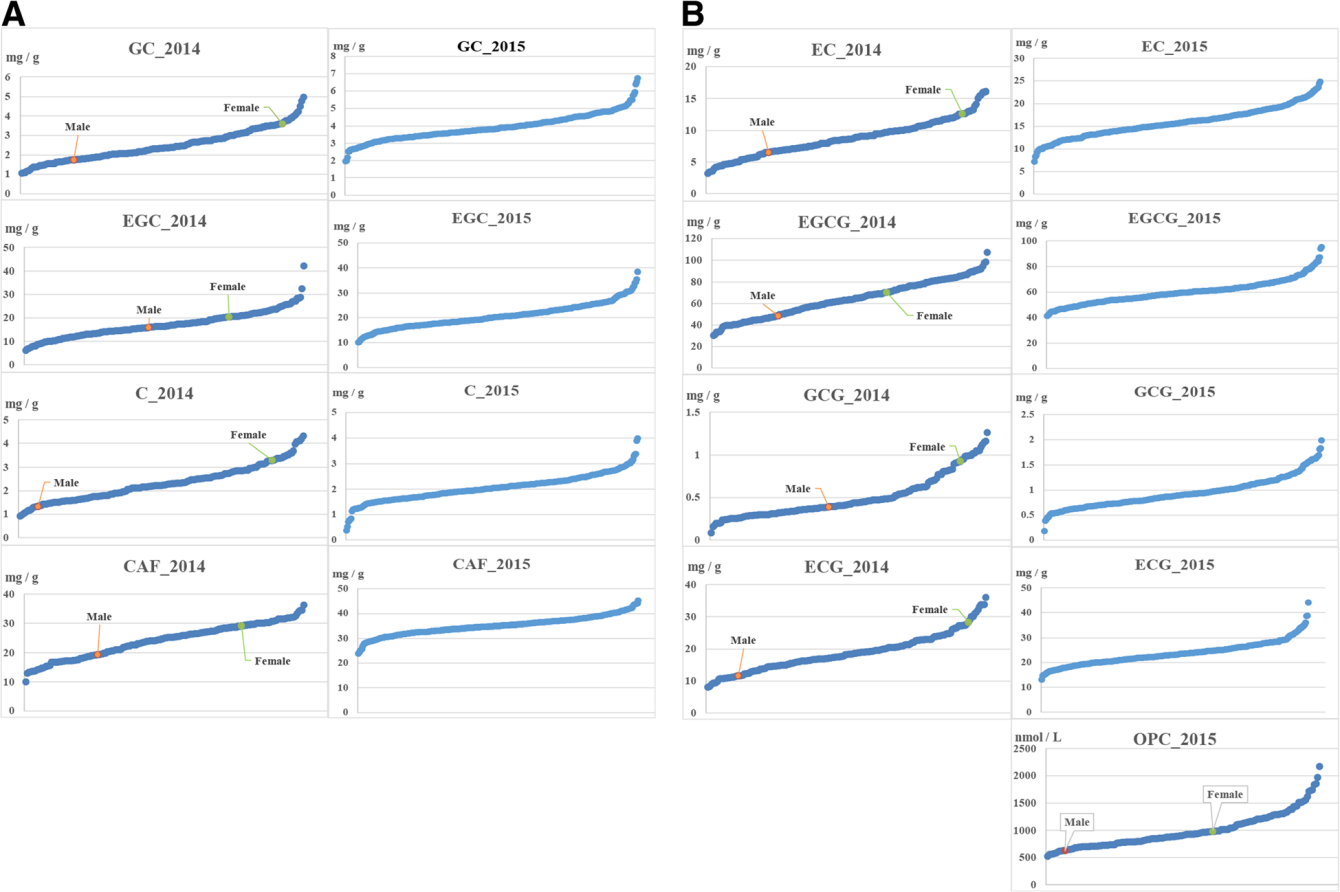
The phenotypic distribution of flavonoid-related traits (X axis is 327 progenies and 2 parents)

The variance degrees of the aimed traits among the segregating population and parents was an important parameter for QTL mapping. Thus, values of coefficient of variation (C.V) were focused on afterward statistical analyses. The results showed the C.V of 9 traits ranged from 10.42 % to 50.38 % and almost all of them were above 20 % (Table 3). So, there was a highly dispersed and variance degrees of traits among the segregating F1 population. Interestingly, we could find the values of C.V showed a substantial reduction from 2014 to 2015 year in each catechin components and CAF, which meant it existed deep environmental impact for different years. Even so, the distribution of F1 progenies was still discrete in each trait, but the range of variation was smaller (Fig. 2). Moreover, the Fig. 2 also displayed the great variation between two parents in flavonoid concentration and CAF.

**Table 3:** The statistical analysis of phenotypic data

Trait ^a^	Min	Max	Range	Mean	Std.E	Std.D	C.V (%)
OPC_2015	515.95	2169.53	1653.58	980.20	24.79	312.54	31.89
GC_2014	1.05	4.98	3.92	2.47	0.06	0.80	32.30
GC_2015	1.97	6.75	4.78	3.91	0.04	0.74	18.94
C_2014	0.92	4.32	3.4	2.30	0.06	0.73	31.85
C_2015	0.38	3.98	3.6	2.05	0.03	0.51	24.66
EGC_2014	6.12	42.09	35.97	17.10	0.41	5.49	32.09
EGC_2015	10.09	38.46	28.37	20.63	0.27	4.84	23.44
CAF_2014	10.03	36.23	26.19	24.05	0.43	5.70	23.71
CAF_2015	23.82	45.29	21.47	35.10	0.20	3.66	10.42
EC_2014	3.18	16.09	12.91	8.70	0.21	2.81	32.33
EC_2015	7.2	24.83	17.63	15.85	0.18	3.24	20.46
EGCG_2014	30.09	107	76.91	63.81	1.26	16.70	26.17
EGCG_2015	40.95	95.04	54.09	59.81	0.52	9.24	15.45
GCG_2014	0.08	1.26	1.18	0.49	0.02	0.25	50.38
GCG_2015	0.18	1.98	1.81	0.94	0.02	0.29	30.95
ECG_2014	8.08	36	27.92	18.73	0.44	5.82	31.07
ECG_2015	13.15	44.05	30.9	23.61	0.26	4.57	19.35

^a^The unit of OPC_2015 is nmol / L, and other traits are all mg / g

### QTL mapping

In our study, regions with LOD ≥ 3 for each LG were identified as major QTLs. Thus, a total of 27 QTLs associated with ten traits were mapped to eight different linkage groups: LG01, LG03, LG06, LG08, LG10, LG11, LG12, and LG13 (Table 4, Additional file 9: Figure S3). Among these QTLs, 4 for OPC were identified on LG03 and LG08, with an R^2^ ranging from 20.3% (qOPC03a) to 42.0% (qOPC08a). Interestingly, 3 QTLs for YSC that were closely connected to the 4 QTLs for OPC were also mapped to LG03 and LG08. Additionally, the same closely related marker (f707) was found at the peak of qOPC08a and qYSC08a. It also increased at the peak of qOPC03a and qYSC03a (marker dm58). Moreover, three QTLs of OPC (qOPC08a, qOPC08b, qOPC03a) had an overlapping region with those of YSC (qYSC08a, qYSC08b, qYSC03a) on the genetic map. Among these QTLs, 2 of YSC were reported in previous studies, and 1 new QTL (qYSC08b) was identified for the first time in this study.

**Table 4:** QTLs detected in the F1 population

Traits	QTL name	LGs	Position	Marker ^a^	LOD ^b^	R^2^ %^c^
OPC	qOPC03a_2015	LG03	112.82	dm58	7.4	20.3
	qOPC08a_2015	LG08	14.70	f707	17.24	42.0
	qOPC08b_2015	LG08	33.92	f1360	11.8	29.7
	qOPC08c_2015	LG08	58.24	df710	9.87	27.1
YSC	qYSC03a_2015	LG03	112.82	dm58	10.75	14.7
	qYSC08a_2015	LG08	14.70	f707	38.94	42.8
	qYSC08b_2015	LG08	33.07	f430	28.15	33.5
GC	qGC01a_2014	LG01	74.15	F1147	3.82	9.8
	qGC01a_2015	LG01	76.62	df487	9.79	13.5
	qGC10a_2015	LG10	23.95	f369	6.67	9.4
	qGC10b_2015	LG10	40.77	h302	6.94	9.7
	qGC10c_2015	LG10	59.29	h171	7.37	10.5
EGC	qEGC03a_2014	LG03	121.20	h90	3.70	9.6
	qEGC03a_2015	LG03	94.41	f908	7.07	10.1
	qEGC06a_2015	LG06	59.03	f1192	10.56	14.3
	qEGC10a_2014	LG10	43.37	dm629	5.38	13.9
	qEGC10a_2015	LG10	53.49	f951	6.00	8.4
C	qC12a_2014	LG12	0.00	m997	4.34	15.2
	qC12a_2015	LG12	14.67	f730	7.03	9.9
CAF	qCAF11a_2014	LG11	91.90	dm870	4.04	15.7
	qCAF11b_2015	LG11	35.34	f983	5.74	9.2
EC	qEC03a_2015	LG03	94.47	h41	5.40	7.8
	qEC06a_2015	LG06	64.25	dm16	6.72	9.5
	qEC10a_2014	LG10	39.81	h188	4.11	10.6
	qEC13a_2014	LG13	75.27	h343	3.96	10.3
	qEC13a_2015	LG13	96.74	f1003	6.44	10.3
EGCG	qEGCG01a_2015	LG01	110.87	m1038	5.28	7.7
	qEGCG06a_2014	LG06	42.35	f654	4.52	11.5
	qEGCG06a_2015	LG06	49.3	df326	8.68	11.9
	qEGCG06b_2014	LG06	64.25	dm16	5.24	13.7
	qEGCG06b_2015	LG06	58.47	f661	8.33	11.5
GCG	qGCG11a_2015	LG11	10.32	f1859	7.85	18.9
ECG	qECG06a_2014	LG06	63.96	df885	3.14	8.2
	qECG06b_2015	LG06	45.97	f881	5.29	7.5
	qECG12a_2014	LG12	56.54	m527	3.15	8.3
	qECG12a_2015	LG12	64.6	df676	6.13	9.1

^a^Indicates the closely linked marker in the LOD peak of QTL

^b^Indicates the logarithm of odds score

^c^Indicates the percentage of phenotypic variation explained

Both two QTLs for CAF were identified on LG11, but they were in different positions in 2014 and 2015. The major QTL, qCAF11a, was located at 91.90 cM with the largest R^2^ (15.7%), and the marker dm870 was close to it. The other two QTLs with a small R^2^ were located at 35.34 cM (9.2%).

A total of 17 QTLs associated with seven components of catechins were mapped to 7 different linkage groups containing LG01, LG03, LG06, LG10, LG11, LG12, and LG13. Among these QTLs, 6 major QTLs of 4 components (qEGC06a, qEC06a, qEGCG06a, qEGCG06b, qECG06a, qECG06b) were primarily clustered into regions from 40 cM to 60 cM on LG06. The LOD of each QTL was relatively higher and ranged from 3.14 to 10.56. Remarkably, qEGCG06a and qEGCG06b were stable, and the major QTLs found via 2-year testing, except for qECG06a and qECG06b, were very close, although they had no overlaps. Another QTL cluster was in the region from 35 to 60 cM in LG10. The QTLs of the GC were primarily clustered on this region, except for qGC01a, with an R^2^ from 9.4 to 10.5, and one stable QTL of EGC (qEGC10a) was also identified in this region.

Two stable QTLs (qC12a, qECG12a) were distributed in different regions in LG12, with an R^2^ from 8.3 to 15.2. Moreover, the other stable QTLs were distributed in LG01, LG03, LG07, LG11, and LG13; the number of QTLs in each LG varied from one to two; and the R^2^ values for these QTLs ranged from 7.7 (qEGCG01a) to 18.9 (qGCG11a).

### Marker annotation and candidate genes

A total of 878 mapped markers were successful located in the reference genome, and 634 of them were located to the annotated regions (Additional file 10: Table S6, Additional file 11: Figure S4). Furthermore, based on QTL mapping, 86 markers were found in the QTL regions of ten flavonoid-related traits. To obtain a deep understanding of which markers in the QTL regions were related to flavonoids, we performed functional annotation and found that 64 QTLs had obtained annotation information. Of note, the gene functions that corresponded to 2 markers were considered related to the QTL (Table 5, Additional file 12: Table S7). Marker f707 at the peak LOD value for qOPC08a and qYSC08a has a basic helix-loop-helix domain, which may be a part of MYB-bHLH-WD40. MYB-bHLH-WD40 was confirmed to regulate anthocyanin biosynthesis in plants [40]. Marker f252 in qECG12a has been annotated as chalcone-flavanone isomerase, which is an essential enzyme of the flavonoid synthesis pathway [41, 42].

**Table 5:** Information on the candidate marker/gene related to flavonoid function

Marker	Gene name	LG	Position	Description
f707	CSA000778	LG08	14.70	Basic helix-loop-helix (bHLH) domain
f252	CSA005342	LG12	52.99	Chalcone-flavanone isomerase

## Discussion

### 2b-RAD is a suitable method for genotyping

For SNP genotyping, among sequencing technologies with sophisticated tools, such as Stacks, RAD is the most widely applied method to date [43]. However, it is difficult to make a parallel for high-throughput sample processing due to the labour-intensive and time-consuming nature of creating RAD libraries [44]. A notable feature of 2b-RAD sequencing is the tunable genome representation from libraries by using less degenerate adaptors, such as 5’-NNA-3’ overhangs, which can significantly reduce the cost and workload with an acceptable accuracy [45]. Thus, in contrast to the traditional genotyping method, 2b-RAD is more cost effective and flexible and has been successfully used for developing SNP markers, genotyping and constructing genetic maps. For instance, Cui et al. presented a high-density linkage map that covers 98.5% of the genome with 73 linkage groups using 2b-RAD, and they revealed a ZW-ZZ sex determination system in *Eriocheir sinensis* based on QTL analysis of the gender phenotype [46].

In our research, a large number of SNP markers were successful discovered and genotyped in the F1 population of ‘LJ43×BHZ’ using 2b-RAD. Using high-throughput sequencing and genotyping, a total of 46,932 2b-RAD markers were developed, and of these, 10,816 co-dominant and 2,630 dominant 2b-RAD markers were polymorphic. Among these markers, 4463 markers that passed the χ2 test were selected to construct a genetic linkage map. Due to massive sequencing and strict filtration, the marker quality and quantity conformed to the requirements for constructing a genetic map. Therefore, our study further indicates that 2b-RAD is a suitable method for genotyping tea plants.

### Mapping strategy and population construction

The high self-incompatibility of tea plants [5] it makes it difficult to construct a common population for genotyping and QTL analysis, such as an F2 population, RIL population, and NIL population. Thus, we chose an alternative method to construct an F1 population for genotyping based on a ‘pseudo-testcross’ mapping strategy. This strategy considers that many loci in the cross between two heterozygous individuals will be heterozygous in one parent and null in the other and will segregate 1:1 in their F1 progeny following a testcross configuration [31]. Therefore, the gametic segregation in each individual can be directly and efficiently analysed, and this strategy has been widely applied to construct an F1 population in woody perennials [47]. In our study, two cultivars, LJ43 and BHZ, were selected to construct the F1 population; both are national tea cultivars with different advantages. LJ43 is one of the main cultivars for the product ‘Longjing Tea’, which has unique flavours, whereas early sprouting and high yield are features of BHZ.

In the current research, it was confirmed that the population size can affect the accuracy of genetic maps and QTL analysis, and as the population size increases, the map is more precise [48]. In addition, more than 200 individuals is considered a sufficient quantity for constructing a genetic map [49]. However, it is difficult to construct a sufficient F1 population because the hybridization rate in tea plants is relatively low by both natural and artificial methods. To our knowledge, the population size of all published genetic maps has been from 46 to 183 in tea plants, which may be insufficient for constructing genetic maps and lead to distortion. Through massive hybridization between LJ43 and BHZ, we obtained a relatively large-scale F1 population containing 327 individuals, which provided a higher accuracy for population size.

### High-density genetic map and QTL mapping

In this paper, we present the densest genetic map for a tea plant published thus far. Our map covered 1678.52 cM with an average distance of 0.4 cM between adjacent markers, which is the smallest average distance reported for tea plants. Furthermore, spanning the total genetic map, the gap in more than 98% of adjacent markers was less than 2 cM, indicating that most markers were equally and densely distributed in the genetic map. In addition, compared with the framework map by SSR markers in our previous study, the marker order was almost identical between the two maps, indicating that the high-density map developed here was accurate and that the gaps in the framework map were filled by these newly SNP markers. Thus, our map will be a useful tool for marker-assisted breeding, QTL mapping of important agricultural traits and identification of functional genes.

In this study, 27 major QTLs associated with flavonoids were identified in LG01, LG03, LG06, LG08, LG10, LG11, LG12, and LG13, 7 of which were related to the traits of OPC and YSC. Of note, the major QTLs were in LG03 and LG08 because the QTLs of two traits were identified in the same 3 regions with a high LOD value (7.4 to 38.94). Among them, 3 major QTLs of YSC were reported in our previous study [26]. The OPC is a key factor affecting the colour of young buds. Thus, there are key functional genes for the synthesis of anthocyanin in the abovementioned regions. Moreover, qEC03a, qEGC03a, qEGC10a and qECG12a were identified in this study and were located at the same position as qEC3_2010, qEGC3_2010, qEGC10_2010 and qECG12_2010 in a previous study [18]. Additionally, 13 other QTLs related to catechin components were found to be novel, and no similar position was identified in previous studies. Notably, 6 novel QTLs (qEGC06a, qEC06a, qEGCG06a, qEGCG06b, qECG06a, qECG06b) of 4 traits were clustered in the near or overlapping region in LG06, and 5 QTLs (qEC10a, qEGC10a, qGC10a, qGC10b, qGC10c) were clustered in LG10. These QTL clusters mean that this region may have some functional genes controlling the metabolism of various catechin components. In addition, a QTL of CAF was located for the first time, and two major QTLs were identified in LG11 explaining 9.2 to 15.7% of the phenotypic variation.

### Two candidate marker/genes in LG08 and LG12

Based on QTL analysis and function annotation, two markers, f707 and f252, were selected as the markers most likely to be involved in the metabolism of flavonoids. Firstly, anthocyanins are produced by a specific branch of the flavonoid pathway. The marker f707/CSA000778 gene has a domain that is predicted to encode transcription factor bHLH, which plays important roles in various developmental processes of eukaryotes, and one of the function is to participate in anthocyanin biosynthesis. bHLH family members have a basic helix-loop-helix domain that Lc was the first plant bHLH protein to be isolated[50]. In the Arabidopsis, anthocyanin biosynthesis genes are divided into two subgroups: early biosynthesis genes (EBGs) are activated by co-activator independent R2R3-MYB transcription factors, whereas late biosynthesis genes (LBGs) require an MBW complex which is a ternary complex to activity the anthocyanin biosynthesis genes[51]. The ternary complex consists of MYB, bHLH, and WD40, which is a key transcription factor in the regulation of anthocyanin synthesis in most species. Then, f707 was directly mapped at the peak LOD value of qOPC08a and qYSC08a, which are QTLs for anthocyanin-related traits. Thus, we assume that the encoded protein (bHLH) of the marker f707/CSA000778 gene may be a part of MYB-bHLH-WD40, and this candidate marker/gene is worthy of further investigation. The marker f252/CSA005342 gene was annotated as having the function of chalcone-flavanone isomerase (CHI), which is one of the key enzymes that regulates flavonoid biosynthesis. As we know, chalcone synthase (CHS) and CHI work on the early stages of flavonoid biosynthesis. In the tea plant, coumaroyl-CoA and malonyl-CoA were catalysed to chalcone by CHS. And then, chalcone was further transformed into flavanone by CHI, which can derivate to almost all of the flavonoid compounds [52, 53]. Thus, this candidate gene should be investigated in more detail for verification in a further study.

## Conclusions

We genotyped a large number of F1 individuals (327) from tea plants (LJ43×BHZ) using 2b-RAD sequencing. A high-density genetic map of the tea plant was constructed with 4217 loci (427 published SSRs and 3800 newly SNPs). To our knowledge, it is the highest density genetic map that was constructed for a tea plant, with an average interval of 0.40 cM between adjacent markers in this study. Furthermore, this map was used to identify the QTLs of ten traits related to flavonoids in the tea plant. Finally, 27 QTLs distributed in 8 LGs were identified. In addition, two markers were selected as candidate genes that may be involved in flavonoid metabolism. The present study provides important information for gene discovery, marker-assisted selection breeding and map-based cloning for functional genes related to the flavonoid content in tea plants.

## Additional files

Additional file 1: **Table S1.** The statistics of sequencing results (the adaptors consisted of recognition base and overhang). (XLSX 34 kb)

Additional file 2: **Table S2.** The sequencing depth of 2b-RAD. (XLSX 31427 kb)

Additional file 3: **Table S3.** The information on SNP loci (the ref sequences of 33 bp tag had been removed 3’ sticky end). (XLSX 1788 kb)

Additional file 4: **Table S4.** The genotyping in the F1 population. (XLSX 15834 kb)

Additional file 5: **Table S5.** The details of female and male genetic maps. (XLSX 10 kb)

Additional file 6: **Figure S1.** The genetic map of the F1 population (the Co-dominate markers were named: ‘h’- ‘: hk×hk’, ‘f’- ‘: lm×ll’, ‘m’- ‘: nn×np’, the dominate markers were named: ‘df’- ‘: lm×ll’, ‘dm’- ‘: nn×np’). (ZIP 203345 kb)

Additional file 7: **Table S8.** The position information of SNP markers in the linkage group. (XLSX 170 kb)

Additional file 8: **Figure S2.** A comparison of current and previous genetic maps (map 1 is our SNP genetic map, map 2 quoted from previous study). (ZIP 210362 kb)

Additional file 9: **Figure S3.** The QTLs of ten flavonoid-related traits. (TIF 939 kb)

Additional file 10: **Table S6.** The results of alignment between the genetic map and the reference genome. (XLSX 121 kb)

Additional file 11: **Figure S4.** The annotated markers in the genetic map. (TIF 1089 kb)

Additional file 12: **Table S7.** The marker annotation of the genetic map. (XLSX 26 kb)

## Abbreviations

C: Catechin
CAF: Caffeine content
cM: CentiMorgans
EC: Epicatechin
ECG: Epicatechin gallate
EGC: Epigallocatechin
EGCG: Epigallocatechin gallate
GC: Gallocatechin
GCG: Gallocatechin gallate
LOD: Logarithm of odds
OPC: Anthocyanin content
QTL: Quantitative trait locus
SNP: Single nucleotide polymorphism
SSR: Simple sequence repeat
YSC: Young shoot colour
