# Supplementary figures and images for: High-density SNP linkage map construction and QTL mapping for flavonoid-related traits in a tea plant (Camellia sinensis) using 2b-RAD sequencing
Source: BMC Genomics. 2018 Dec 22;19:955. doi: 10.1186/s12864-018-5291-8 (PMC6304016; doi:10.1186/s12864-018-5291-8)

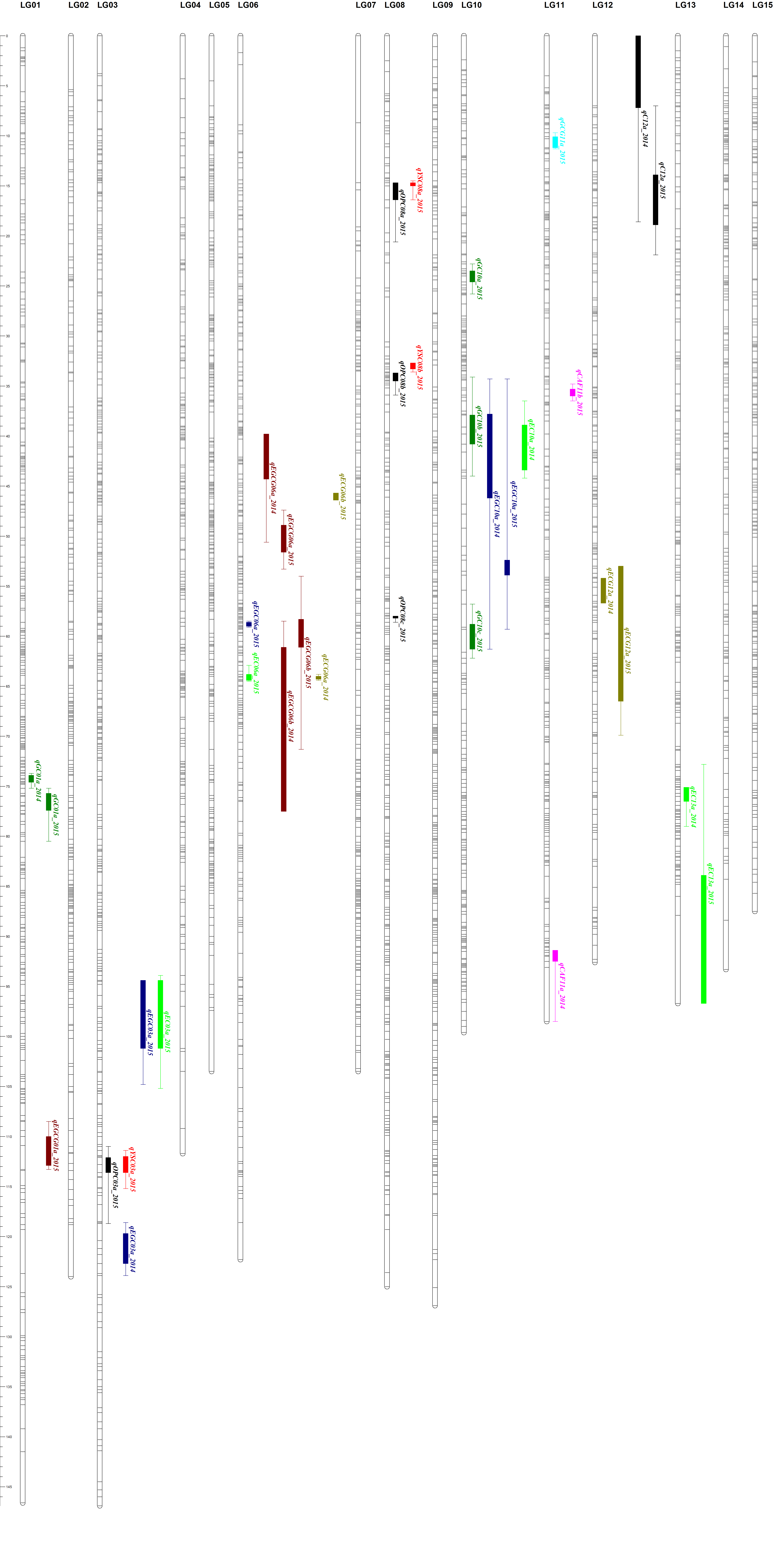

Supplement: Supplementary file 9 — Figure S3. The QTLs of ten flavonoid-related traits. (TIF 939 kb) [file 12864_2018_5291_MOESM9_ESM.tif]

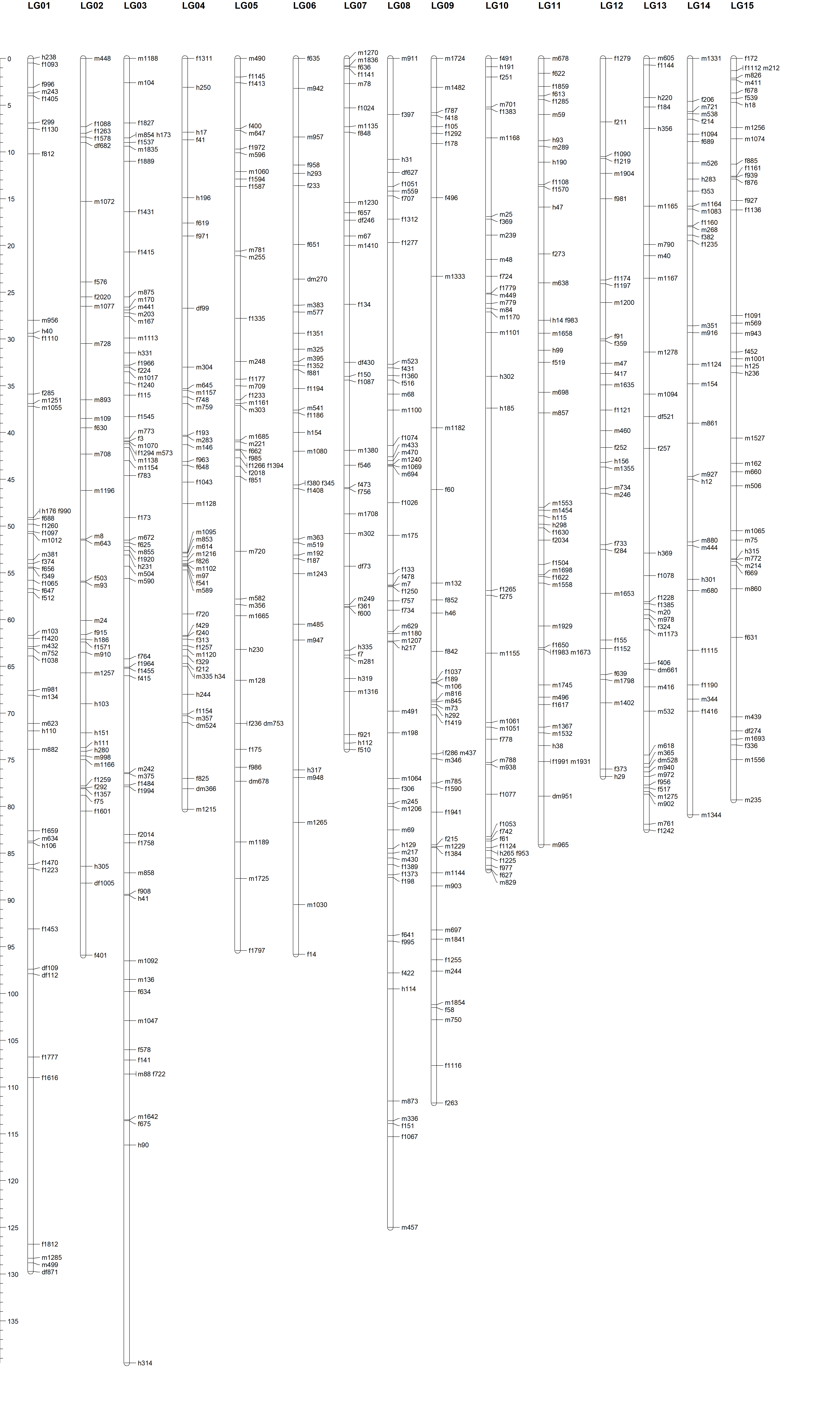

Supplement: Supplementary file 11 — Figure S4. The annotated markers in the genetic map. (TIF 1089 kb) [file 12864_2018_5291_MOESM11_ESM.tif]
